# The complete chloroplast genome of *Castanopsis carlesii* (Hemsl.) Hay

**DOI:** 10.1080/23802359.2019.1641437

**Published:** 2019-07-17

**Authors:** Rong-Xi Sun, Xue-Min Ye, Zhen-Li Wang, Xiao-Fan Lin

**Affiliations:** Jiangxi Provincial Key Laboratory of Silviculture, College of Forestry, Jiangxi Agricultural University, Nanchang, China

**Keywords:** *Castanopsis carlesii*, complete chloroplast genome, phylogenetic analysis

## Abstract

*Castanopsis carlesii* (Hemsl.) Hay. is a widely distributed and dominant tree species with significant ecological and economical values. In this study, the complete chloroplast genome sequence of *C. carlesii* was reported using the Illumina Hiseq 2500 platform. The complete chloroplast genome was 160,205 bp forming a typical quadripartite structure, with a pair of inverted repeated (IRs) regions of 25,670 bp, a large single copy (LSC) region of 89,849 bp, and a small single copy (SSC) region of 19,016 bp. A total of 124 functional genes were annotated, including 79 protein-coding genes, 37 tRNA genes, and eight rRNA genes. The ML phylogenetic analysis showed that the genus *Castanopsis* formed a clade except *Castanopsis fargesii*.

*Castanopsis carlesii* (Hemsl.) Hay. is a widely distributed and dominant tree species of the genus *Castanopsis* from the family Fagaceae in subtropical evergreen broadleaved forest of China, with significant ecological and economical values (Lin et al. 2017). However, few studies on the chloroplast genome of *C. carlesii* was reported, so, we assembled and characterized its complete chloroplast genome, in order to provide information for the study the origin of *C. carlesii.*

The plant material was collected in Ganzhou of Jiangxi Province, China (24°33′N, 114°25′E). The voucher specimen was deposited in Jiangxi agricultural university college of forestry (No. JXMIZHU1). The total genomic DNA of *C. carlesii* was extracted using the Plant Genomic DNA Kit and sequenced using Illumina Hiseq2500 platform. Approximately 6 GB of clean data were yielded. These trimmed reads were assembled by NOVOPlasty (Dierckxsens et al. 2017), and then the assembled genome was annotated using CpGAVAS (Liu et al. 2012) and DOGMA (Lohse et al. 2007).

The complete chloroplast genome of *C. carlesii* was 160,205 bp in length forming a typical quadripartite structure, with a pair of inverted repeated (IRs) regions of 25,670 bp, a large single copy (LSC) region of 89,849 bp, and a small single copy (SSC) region of 19,016 bp. The GC content of the whole chloroplast genome was 36.82%. The proportions of GC contents in IRs, LSC, and SSC were 42.8%, 34.67%, and 30.82%, respectively. A total of 124 functional genes were annotated, including 79 protein-coding genes, 37 tRNA genes, and eight rRNA genes. The majority of genes were single copy, whereas 19 functional genes were duplicated, including eight protein-coding genes, four rRNA genes, and seven tRNA genes. All genes were encoded by 25 997 codons. The annotated chloroplast genome of *C. carlesiii* has been deposited in GenBank with accession number MK745999.

Eight of the genus of *Castanopsis* and *Castanea* chloroplast genome sequences were aligned by MAFFT (Kazutaka et al. 2005). *Corylus heterophylla* was used as the outgroups. Phylogenetic analysis was computed based on the alignment sequences chloroplast genomes by maximum likelihood (ML) analysis by RAxML based on Kimura 2-parameter model with 1000 bootstrap replicates (Alexandros et al. 2017). As shown in the ML phylogenetic tree (Figure 1), the genus *Castanea* formed a monophyletic clade with high bootstrap value, whereas, the genus *Castanopsis* formed a clade except *Castanopsis fargesii*. These results on complete chloroplast genome reported here will lay a basis for the study of phylogeny, phylogeography and population genetic diversity of *C. carlesiii.*

**Figure 1.**
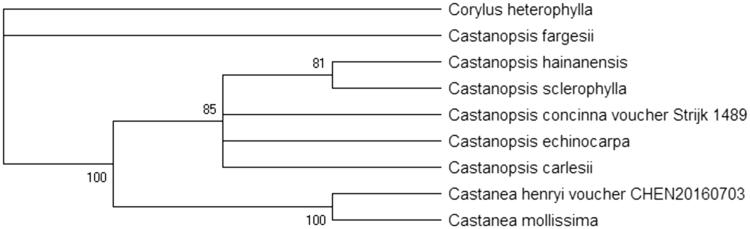
The ML phylogenetic tree based on nine complete chloroplast genomes. Accession number: *Corylus heterophylla* (KX822769), *Castanea henryi* voucher CHEN20160703 (KX954615), *Castanea mollissima* (HQ336406), *Castanopsis concinna* voucher Strijk 1489 (KT793041), *Castanopsis echinocarpa* (KJ001129), *Castanopsis hainanensis* (MG383644), *Castanopsis fargesii* (MK571045), *Castanopsis carlesii* (MK745999), and *Castanopsis sclerophylla* (MK387847). The number on each node indicates the bootstrap value.
